# Concomitant Expression of Prolactin Receptor and TGFβ Receptors in Breast Cancer: Association with Less Aggressive Phenotype and Favorable Patient Outcome

**DOI:** 10.3390/ijms20071640

**Published:** 2019-04-02

**Authors:** Ibrahim Y. Hachim, Vanessa M. López-Ozuna, Mahmood Y. Hachim, Jean-Jacques Lebrun, Suhad Ali

**Affiliations:** 1 Department of Medicine, McGill University Health Center, Cancer Research Program, Montreal, QC H4A 3J1, Canada; ibrahim.hachim@mail.mcgill.ca (I.Y.H.); vanessa.lopez2@mail.mcgill.ca (V.M.L.-O.); jj.lebrun@mcgill.ca (J.-J.L.); 2 Sharjah Institute for Medical Research, University of Sharjah, P.O. Box 27272, Sharjah, UAE; u16101425@sharjah.ac.ae

**Keywords:** epithelial–mesenchymal transition, differentiation, prognosis, co-expression

## Abstract

The epithelial–mesenchymal transition (EMT) process is known to play an essential role in tumor progression, metastasis and resistance to therapy. This report evaluated the prognostic value of co-expression of the receptor for prolactin (PRLR), a suppressor of EMT, and the receptors for transforming growth factor β (TGFβRI and TGFβRII), an inducer of EMT, in association with different clinicopathological parameters using TMA of 102 breast cancer patients and publicly available data on breast cancer patients. Interestingly, the results revealed that malignant tissues had significantly lower levels of concomitant protein expression of these receptors in comparison to normal/benign breast tissue. In addition, a higher level of concomitant expression was also observed in less aggressive breast cancer phenotypes, including low grade tumors, luminal breast cancer subtype, and less advanced stages of the disease (lymph node negative and early stages). Moreover, the results also showed that the expression of a gene signature composed of PRLR/TGFβRI/TGFβRII correlates more with differentiated grade I tumors, and identified a subset of patients showing better survival outcomes evident in luminal B and HER-2 enriched molecular subtypes. Together, these results indicate that loss of the co-expression of PRLR, TGFβRI and TGFβRII is indicative of aggressiveness and poor patient survival outcomes in breast cancer.

## 1. Introduction

Cancer cells not only exhibit the loss of normal mechanisms regulating growth, but also alterations in their differentiation program defined as cellular plasticity, allowing the acquisition of phenotypic changes associated with features of aggressive cancer [1,2]. Clinically, major challenges in the management of cancer have been linked to the plasticity of cancer cells, including tumor heterogeneity, metastasis, recurrence and the development of resistance to therapy. The epithelial–mesenchymal transition (EMT) process, important in development and wound healing and assumed by cancer cells, has been asserted as the molecular mechanism leading to cancer cell plasticity. Major challenges still exist in the clinical application of the EMT process regarding patient prognosis, survival outcome and response to therapy [3]. Therefore, the characterization of regulators of the EMT process and evaluating their clinical significance may offer new information for patient prognosis and targets for developing new therapies against metastasis, recurrence and drug resistance that target the EMT process.

In breast cancer, transforming growth factor β (TGFβ) is believed to play important roles ranging from tumor suppression to tumor promotion [4]. While the tumor suppressor roles were found to be restricted to the normal, premalignant as well as early breast carcinomas, in more advanced stages of the disease, TGFβ is well known for promoting EMT, contributing to cancer progression and metastasis [4,5,6,7]. Clinically, tumors with more aggressive phenotypes and high grades are usually characterized by increased TGFβ production [8,9]. In addition, within invasive carcinoma samples, the expression of TGFβ receptors (TGFβRI and TGFβRII) were found to be increased with disease progression, advanced tumors, as well as more aggressive phenotype [10]. On the other hand, the hormone prolactin (PRL) is known for its crucial role in mammary gland development and the terminal differentiation of mammary epithelial cells [11,12]. While the role of PRL in breast cancer has yet to be fully defined, PRL and its signaling pathway Jak2/Stat5 were shown to stabilize cell adhesion complexes and suppress the EMT process and invasive properties of breast cancer cells [13,14,15]. In addition, using large cohorts of human breast cancer cases, PRL receptor (PRLR) expression was found to be downregulated during tumor progression, from benign, in situ to invasive carcinoma [16]. Furthermore, PRL, PRLR and Stat5 expression levels were found to correlate with favorable clinicopathological parameters and better patient survival outcomes [16,17,18,19,20,21]. Interestingly, a negative cross-talk between TGFβ and PRL pathways has been observed in mammary epithelial and breast cancer cells. Whereas TGFβ/Smad signaling was found to block PRL-induced Stat5 activation, PRL/Jak2 signaling was found to suppress TGFβ/Smad activation [13,22]. However, the clinical value of the relationship between these two pathways in breast cancer prognosis or patient outcome has yet to be investigated.

## 2. Results

### 2.1. Immunohistochemical Analyses of Co-Expression of PRLR and TGFβ Receptors in Relation to Breast Cancer Progression

**Figure 1:**
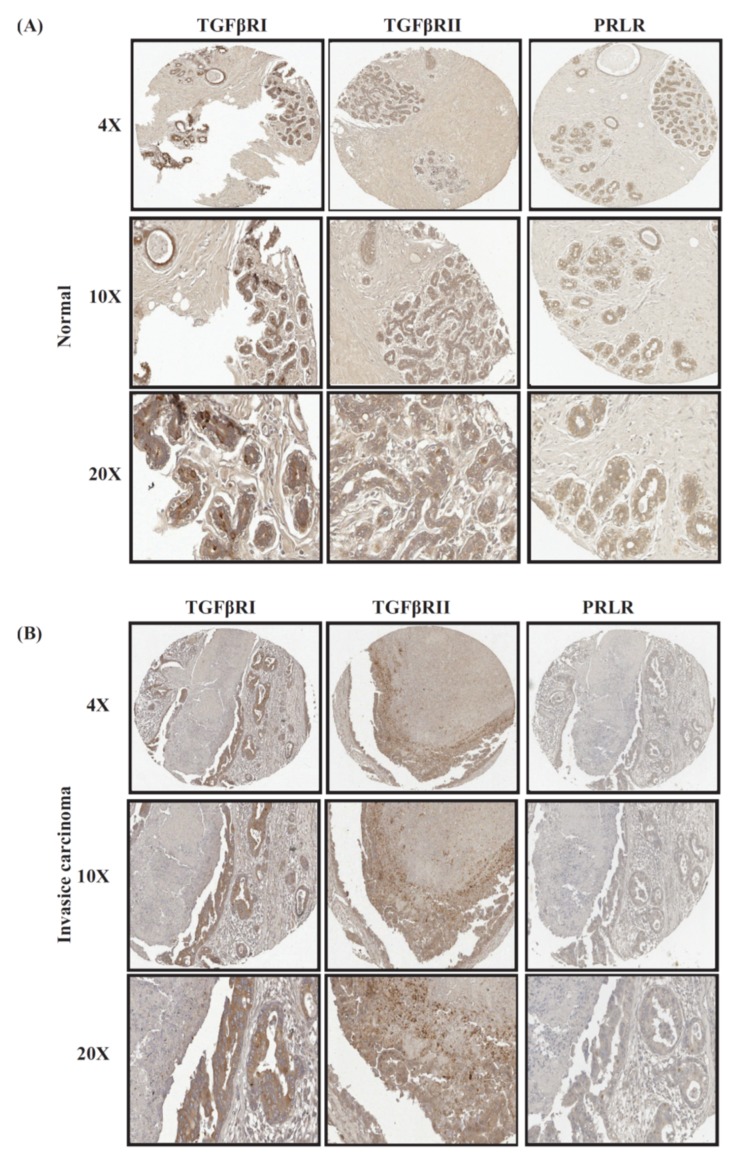
Representative images of co-expression of PRLR with both TGFβ receptors in (**A**) normal/benign samples; (**B**) invasive breast cancer samples.

Previous studies have found a negative cross-talk between PRL and TGFβ pathways in mammary and breast cancer cells. Precisely how the relationship between these two factors manifests clinically is still not known. Earlier studies using a panel of normal breast and breast cancer cases examined the expression of PRLR as well as TGFβRI and TGFβRII individually [16,23]. This study examined the pattern of co-expression of these receptors using the same clinical cases we used in our earlier studies in relation to various clinicopathological parameters. Interestingly, as shown in Table 1 and Figure 1, high levels of co-expression were observed between PRLR and TGFβRI (80%) as well as between PRLR and TGFβRII (75%) in normal breast tissues. The levels of co-expression of these receptors were found to be slightly reduced in the early in situ lesions for PRLR/TGFβRI and PRLR/TGFβRII (60%). Importantly, the extent of co-expression of these receptors in invasive carcinomas significantly declined to only 29.5% for PRLR/TGFβR1 (*p* = 0.02) and 28.12% for PRLR/TGFβR2 (*p* = 0.049) (Table 1, Figure 1). Indeed, in both normal as well as malignant samples, the area that showed positive PRLR staining was, in most cases, also positive for both TGFβ receptors; however, this area was reduced in the malignant samples compared to normal samples. These results highlight the gradual loss of PRLR co-expression with TGFβ receptors during breast tumorigenesis.

Subsequently, the co-expression levels between PRLR and TGFβ receptors were evaluated in cases representing different stages of tumor progression. As can be seen in Table 1, while the state of receptors co-expression showed minimal variation in relation to tumor size, receptors co-expression decreased in relation to lymph node involvement from 38.23% to 28.12% for PRLR/TGFβRI and from 36.11% to 21.21% for PRLR/TGFβRII. Moreover, the results also showed higher levels of co-expression between the two receptor classes in early stage tumors in comparison to advanced stages, from 34.21% to 24% for PRLR/TGFβRI and from 31.57% to 20.68% for PRLR/TGFβRII (Table 1). Together, these results indicate a loss of concomitant expression of PRLR with TGFβ receptors in invasive and advanced stage breast tumors.

### 2.2. Co-Expression Levels of PRLR and TGFβ Receptors in Relation to Tumor Grade in Breast Cancer

**Figure 2:**
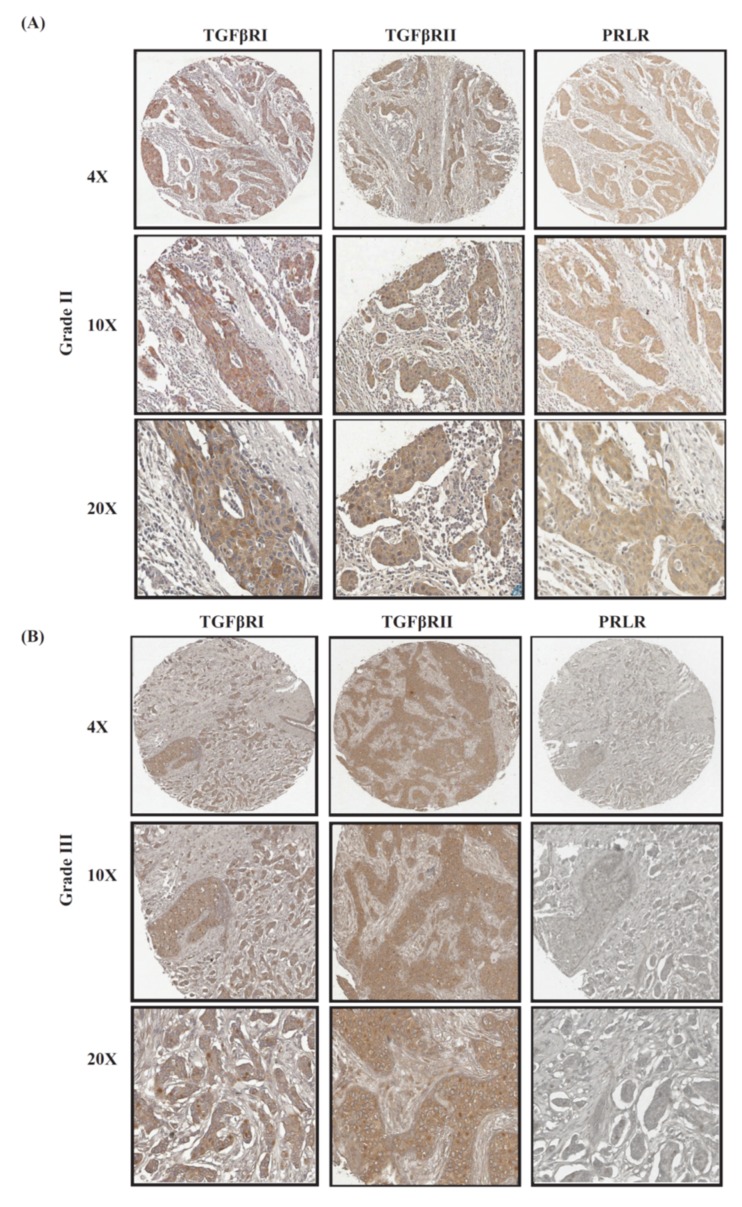
Representative images of co-expression of PRLR with both TGFβ receptors in (**A**) grade II tumors compared to (**B**) grade III tumors.

**Figure 3:**
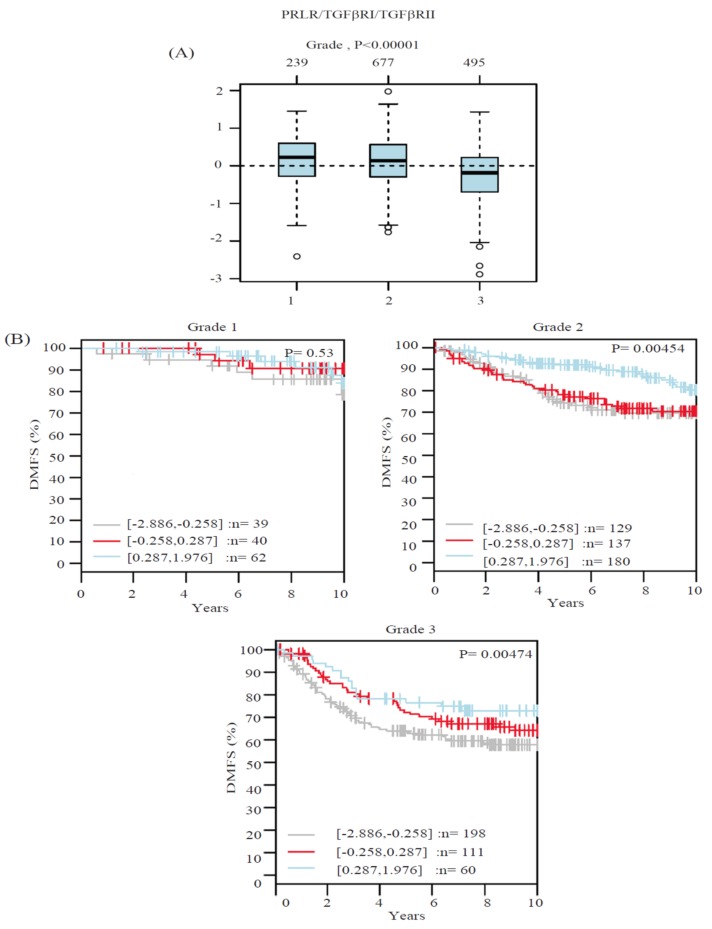
PRLR/TGFβRI/TGFβRII gene signature and its association with tumor grade and patient outcome. (**A**) Boxplot of PRLR/TGFβRI/TGFβRII gene signature in 1881 breast cancer cases from the GOBO database stratified according to histological grade. The band inside the box represents the median. The top and bottom of the box indicate the distance between quartile 1 (Q1), quartile 3 (Q3) and 1.5 times the interquartile range. In addition, the circles (when present) indicate the outliers. (**B**) PRLR/TGFβRI/TGFβRII gene signature and its association with patient outcome using distant metastasis-free survival (DMFS) as an end point in different tumor grades using 1881 breast cancer cases from the GOBO database.

Another important clinicopathological parameter in determining the aggressivity of breast cancer is tumor grade. Therefore, the profile of PRLR and TGFβ receptors co-expression in relation to tumor grade were examined using the same clinical cases used in the tissue microarray (TMA) above. As can be seen in Figure 2 and Table 1, the results revealed that more differentiated (grade I) tumors showed higher levels of co-expression of PRLR/TGFβR1 (20% of cases) and PRLR/TGFβR2 (30% of cases) in comparison to poorly differentiated (grade III) cases, where PRLR/TGFβR1 was reduced to 15.38% and PRLR/TGFβR2 was reduced to 15.38%. This loss of co-expression of PRLR with TGFβ receptors, despite its importance, did not reach significant levels. This can be attributed to the limited number of cases in the cohort. For a better evaluation of this point, the correlation of the co-expression of PRLR and TGFβ receptors in relation to tumor grade was investigated using the Gene expression-based Outcome for Breast cancer Online (GOBO) database, a large publicly available database of 1881 breast cancer cases. Data were generated in relation to a gene signature composed of PRLR/TGFβR1/TGFβRII. Interestingly, as can be seen in Figure 3A, there is a significant loss of expression of the gene signature in high-grade cases in comparison to low-grade breast cancer cases. Subsequently, the correlation between receptors co-expression and the survival outcome for patients in each grade category was evaluated. Importantly, cases with co-expression of the three receptors show significantly better patient survival outcomes, especially in aggressive grade II and grade III cases (Figure 3B). The contribution of co-expression of the three receptors to better survival outcomes in aggressive grades II and III cases was also evident when compared to survival outcomes using a gene signature composed of only the two TGFβ receptors (Figure S1). Together, these results emphasize that co-expression of the three receptors together may identify a distinct patient population with better survival outcomes.

### 2.3. Co-Expression Levels of PRLR and TGFβ Receptors in Relation to Breast Cancer Molecular Subtypes

Due to the heterogeneity of breast cancer and the fact that each breast cancer molecular subtype represents a distinct entity in its clinical, molecular and therapeutic management, the co-expression levels of PRLR with both TGFβ receptors in different breast cancer molecular subtypes were evaluated using the same panel of TMA and the same classification methods used previously [10,16]. As expected, while breast cancer cases of luminal and human epidermal growth factor-2 (HER-2) overexpression subtypes showed concomitant expression of PRLR with TGFβRI and TGFβRII in a range of 27.77% to 34.38% of cases, triple negative breast cancer (TNBC), the most aggressive breast cancer subtype, showed no co-expression between PRLR with either of the two TGFβ receptors (Table 1).

**Figure 4:**
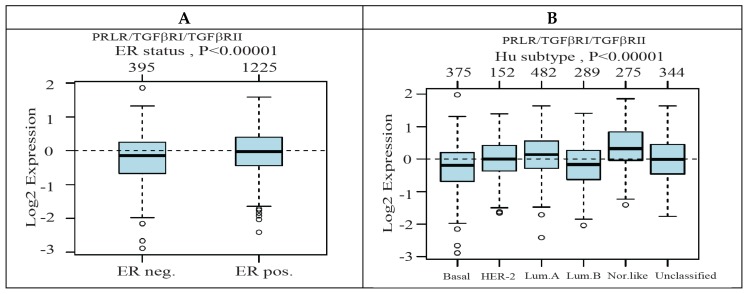
The expression level of PRLR/TGFβRI/TGFβRII gene signature and its association with estrogen receptor (ER) status and breast cancer molecular subtypes. (**A**) Boxplot of PRLR/TGFβRI/TGFβRII gene signature in 1881 breast cancer cases from the GOBO database stratified according to ER status. The band inside the box represents the median and the top and bottom of the box indicate the distance between the quartile 1 (Q1), quartile 3 (Q3) and 1.5 times the interquartile range. The circles indicate the outliers. (**B**) PRLR/TGFβRI/TGFβRII gene signature and its association with breast cancer molecular subtypes (Hu classification) using 1881 breast cancer cases from the GOBO database.

We next compared the expression level of the gene signature of PRLR/TGFβRI/TGFβRII generated above in relation to estrogen receptor (ER) expression, as well as in relation to the different breast cancer molecular subtypes using clinical cases available from the GOBO database. Interestingly, the receptors gene signature were found to be significantly associated with the less aggressive ER-positive tumors compared to ER-negative tumors (*p* < 0.00001) (Figure 4A). This is in agreement with data obtained when the expression of the gene signature was examined in breast cancer cases according to Hu sub-classification. As shown in Figure 4B, the highest level of expression of PRLR/TGFβRI/TGFβRII was found in the luminal A subtype and the lowest expression was found in the basal-like subtype.

### 2.4. Higher Levels of Expression of PRLR/TGFβRI/TGFβRII Gene Signature Are Associated with Prolonged Survival in Luminal B and HER-2-Enriched Breast Cancer Molecular Subtypes

**Figure 5:**
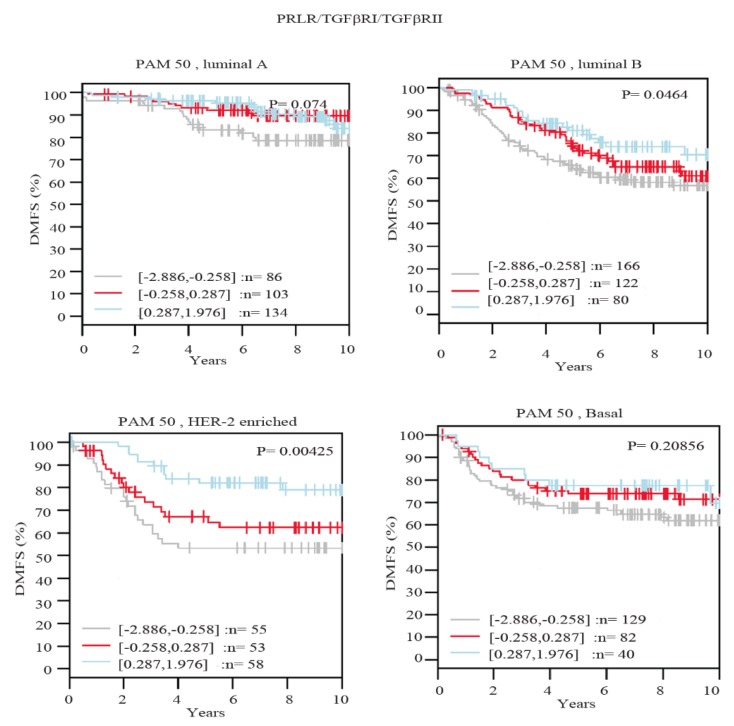
The association between PRLR/TGFβRI/TGFβRII gene signature and patient outcome in different breast cancer subtypes.

To further evaluate the clinical value of PRLR co-expression with TGFβ receptors in breast cancer, the correlation of the expression of the three receptors was examined in relation to patient survival outcomes using distant metastasis-free survival (DMFS) as an endpoint in the GOBO database. Importantly, as can be seen in Figure 5, breast cancer cases showing high expression of the three receptors showed significantly better survival outcomes in HER-2-enriched (*p* = 0.00425) and luminal B (*p* = 0.0464) subtypes. Furthermore, while high co-expression of the three receptors showed better survival outcomes in luminal A and basal-like breast cancer subtypes, this effect did not show a statistical significance. Together, these results emphasize that breast cancer patients that maintain the expression of the three receptors are less likely to develop distant metastasis in comparison to patients showing low expression levels.

## 3. Discussion

Breast cancer heterogeneity, metastasis and resistance to therapy have been linked to the ability of cancer cells to adopt various epithelial/mesenchymal cell states through the EMT process. Although this process is well recognized in laboratory settings, the value of EMT in clinical settings is still debatable. This study examined the correlation between the expression of the receptors for the growth factor TGFβ, an inducer of EMT, and the receptor for PRL hormone, a suppressor of EMT, in relation to clinicopathological criteria and patient outcomes in breast cancer clinical cases. The results showed that concomitant PRLR expression with TGFβ receptors in breast cancer clinical samples was significantly downregulated in cancer samples compared to normal/benign tissue. In addition, the level of co-expression between TGFβ receptors and PRLR was found to decrease with tumor progression, more advanced stages of the disease and lymph node (LN)-positive tumors.

The EMT process is an essential step in cancer cell dissemination and tumor progression. This is mainly attributed to its involvement in various malignant functions including tumor initiation, cell migration, LN involvement, metastasis as well as therapy resistance [24,25]. Indeed, the co-expression between PRLR and both TGFβ receptors was found to be reduced by around 50% in invasive ductal carcinoma cases that are characterized by LN involvement compared with cases with no LN involvement (from ~30% to 15%). This result was further supported by findings that patients characterized by the preservation of high levels of expression of a gene signature composed of PRLR and both TGFβRI and TGFβRII have a lower chance of developing distant metastasis. This association between the preservation of higher levels of the PRLR/TGFβRI/TGFβRII gene signature reached statistical significance in HER-2 as well as luminal B breast cancer subtypes.

In addition, an essential finding in this report was the strong association between high co-expression levels of the three receptors with low-grade tumors, which is one of the best and well-established favorable prognostic markers in breast cancer [26]. Indeed, higher levels of co-expression between TGFβ receptors and PRLR were found to be associated with more differentiated tumors, while low co-expression levels were found in high grade, poorly differentiated tumors. These findings agree with previous reports implicating EMT in cancer biology to be exclusively involved in the dedifferentiation of malignant cells and the enhancement of tumor stemness. Moreover, it also highlights the difference between EMT observed in cancer and EMT observed in development that might play differentiation and/or dedifferentiation roles [27]. Furthermore, the association between the high co-expression level of the TGFβ receptors and PRLR was not restricted to more differentiated tumors and less advanced stages of the disease, but was also associated with other markers of less aggressive phenotypes, including an association with ER-positive tumors and the luminal subtype. In summary, our results may improve our understanding of the complicated interaction between factors involved in the regulation of the EMT process and their prognostic and clinical values.

## 4. Materials and Methods

### 4.1. Tissue Samples

The tissue microarray (BRC1021) purchased from Pantomics (Richmond, CA, USA) was used according to the guidelines of the Research Institute of the McGill University Health Center. The company provided assurances that all tissues were collected under strict ethical guidelines and protocols. In addition, all patients gave written informed consent. This TMA contained five normal/benign cores, in addition to 97 breast cancer samples of different stages and grades. The expression levels of different classical markers, such as estrogen receptor (ER), progesterone receptor (PR), HER-2, Ki67 and P53, were also provided. The tumor samples consisted of six in situ carcinoma and 91 invasive carcinoma samples. Most of the samples were invasive ductal carcinomas (83 cases), while six cases were invasive lobular carcinomas, in addition to one papillary and one mucinous carcinoma. According to grade, 19 cases were classified as grade I, 54 cases as grade II and 16 cases were grade III. In addition, six cases were classified as stage 0 (in situ carcinoma) and 55 cases were stages I and II. There were 36 cases with the advanced stages III and IV.

The classification of cases according to different molecular breast cancer subtypes was performed using immunohistochemistry surrogates of breast cancer molecular subtypes previously described [16,28]. In brief, cases with ER+ and/or PR+, HER-2- with Ki67 < 14% were classified as luminal A. Cases with ER+ and/or PR+, HER-2- with Ki67 > 14% or ER+ and/or PR+ with HER-2+ were classified as luminal B. In addition, cases that showed negative ER and PR with HER-2 positivity were classified as HER-2-positive. Cases that lacked the three receptors (ER, PR and HER-2) were classified as triple-negative. The T47D breast cancer cell line, which represents the luminal A subtype and known to express PRLR, was used as a positive control for PRLR expression. In contrast, the MDA-MB-231 breast cancer cell line, which represents the triple-negative breast cancer subtype, was used as a positive control for both TGFβ receptors.

### 4.2. Immunohistochemistry

Initially, the slides were baked at 55 °C in an oven for 30 minutes and then deparaffinized using xylene. This was followed by the step of rehydration, which included the use of serial dilutions of alcohol. The slides were then subjected to heat-induced antigen retrieval using sodium citrate 10 mM, pH 6.0 buffer. This was followed by incubation for 10 minutes in hydrogen peroxide block, which was followed by Ultra V Block. The slides were then stained with different antibodies including PRLR (Santa Cruz#sc-20992), TβRII (SC-398) and TβRI (SC-220-R). For staining visualization, the UltraVision LP Detection System HRP Polymer and DAP Plus Chromogen (Thermo Fisher Scientific, Fremont, CA, USA) was used.

### 4.3. Immunohistochemistry Scoring

Cases were considered positive for PRLR expression if the malignant cells showed ≥10% of positive membranous and/or granular cytoplasmic staining [16]. Cases were considered positive for TβRI and TβRII expression if the malignant cells showed >20% positive staining [10]. For both ER and PR status, the Allred scoring system was followed as previously described [10]. Moreover, we followed the American Society of Clinical Oncology/College of American Pathologists guidelines for HER-2 scoring with 0 score and +1 score cases considered as negative and +2 score cases considered as equivocal, while only +3 score cases were considered positive. Cases were also considered positive for Ki-67 expression if they showed positive nuclear staining ≥14%.

### 4.4. Data Mining

The GOBO database was used to evaluate the association between PRLR and both TGFβ receptors gene expression levels. The GOBO database provided the ability to evaluate the association between the expression levels of single or multiple genes with different clinicopathological parameters, as well as patient outcomes in 1881 breast cancer patient samples [29]. The Gene Set Analysis (GSA)-Tumor tool in the database allowed the classification of patients according to the expression levels of different genes into equal quantiles to define high, intermediate and low expression. The association between gene expression and tumor grade, ER status and molecular subtypes was presented as a Boxplot, where the band inside the box represents the median and the top and bottom of the box indicate the distance between the Q1, Q3 and 1.5 times the interquartile range. The outliers were presented as circles. For the association between the tumor grade and molecular subtypes, an ANOVA test (computed by the database) was performed to evaluate the levels of significance as previously described [29].

### 4.5. Statistical Analysis

The co-expression levels between PRLR and both TGFβ receptors were tabulated. The association between co-expression levels and the classical pathological as well as clinical variables were evaluated using a chi-square test in the GraphPad Prism6 software.

## 5. Conclusions

This study revealed that the loss of concomitant expression of the PRL and TGFβ receptors occurs during breast cancer progression. In contrast, high expression of a gene signature of PRLR/TGFβRI/TGFβRII was shown to be an indicator of low-grade tumors and a marker of better patient outcomes. Together, these results provide new tools for patient stratification and prognosis in breast cancer.

## Figures and Tables

**Table 1:** The association between the co-expression levels of prolactin receptor and transforming growth factor β receptors (PRLR/TGFβRI and PRLR/TGFβRII) in relation to clinicopathological parameters.

PRLR					PRLR				
TGFβRII-positive cases	Negative	Positive	% co-expression	*p*-value	TGFβRI-positive cases	Negative	Positive	% co-expression	*p*-value
Normal vs. malignant cases									
Normal and benign	1	3	75%	0.049		1	4	80%	0.021
Invasive Ca	46	18	28.12%			43	18	29.50%	
Histological grade									
Grade I	7	3	30%	0.67		8	2	20%	0.72
Grade II	29	10	25.64%			29	10	25.6%	
Grade III	11	2	15.38%			11	2	15.38%	
LN involvement									
LN-negative	23	13	36.11%	0.17		21	13	38.23%	0.38
LN-positive	26	7	21.21%			23	9	28.12%	
Tumor size									
T1 and T2	22	9	29.03%	0.76		21	9	30%	0.71
T3 and T4	26	9	25.71%			23	8	25.80%	
Tumor stage									
Stages 1 and 2	26	12	31.57%	0.31		25	13	34.21%	0.38
Stages 3 and 4	23	6	20.68%			19	6	24%	
Breast cancer subtype									
Luminal	23	10	30.3%	0.29		19	10	34.48%	0.34
HER-2	13	5	27.77%			13	5	27.77%	
TNBC	6	0	0%			6	0	0%	

## Supplementary Materials

Supplementary materials can be found at https://www.mdpi.com/1422-0067/20/7/1640/s1.

## Abbreviations

EMT	Epithelial–mesenchymal transition
PRLR	Prolactin receptor
DMFS	Distant metastasis-free survival
TNBC	Triple-negative breast cancer
HER-2	Human epidermal growth factor receptor 2
TGFβ	Transforming growth factor beta
