# Supplementary material for: Concomitant Expression of Prolactin Receptor and TGFβ Receptors in Breast Cancer: Association with Less Aggressive Phenotype and Favorable Patient Outcome
Source: Int J Mol Sci. 2019 Apr 2;20(7):1640. doi: 10.3390/ijms20071640 (PMC6479350; doi:10.3390/ijms20071640)
Supplement: Supplementary file 1 [file ijms-20-01640-s001.pdf]

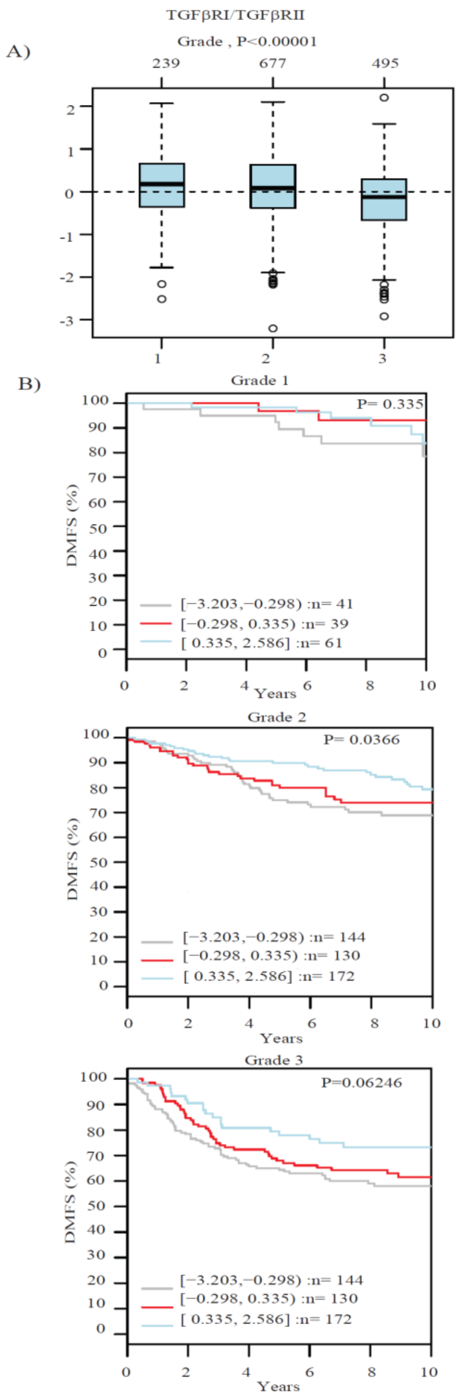

Figure Supplemental 1: TGFβRI/TGFβRII gene signature and its association with tumor grade and patient outcome

A) TGFβRI/TGFβRII gene signature and its association with tumor grade using1881 breast cancer cases of GOBO database.

B) TGFβRI/TGFβRII gene signature and its association with patient outcome using DMFS as an end point in different tumor grades using 1881 breast cancer cases of GOBO database.
